# A Study to Assess the Dosimetric Impact of the Anatomical Changes Occurring in the Parotid Glands and Tumour Volume during Intensity Modulated Radiotherapy using Simultaneous Integrated Boost (IMRT‐SIB) in Head and Neck Squamous Cell Cancers

**DOI:** 10.1002/cam4.4079

**Published:** 2021-06-22

**Authors:** Arunima Ghosh, Seema Gupta, Danial Johny, Vivek Vidyadhar Bhosale, Mahendra Pal Singh Negi

**Affiliations:** ^1^ Department of Radiotherapy King George’s Medical University Uttar Pradesh Lucknow India; ^2^ Toxicology and Experimental Medicine Division CSIR‐Central Drug Research Institute Lucknow India

**Keywords:** adaptive radiation, dosimetric variation, head neck cancer, imrt, parotid gland, volumetric variation

## Abstract

**Background:**

Anatomical variations in head and neck cancer during IMRT leads to volume shrinkage, results in dosimetric variations in tumour and normal tissue including parotid glands, with a risk of radiation toxicities.

**Methods:**

30 patients with a stage II–IV head and neck squamous cell carcinoma (HNSCC) were treated with definitive IMRT‐SIB and concomitant chemotherapy. Volumetric and dosimetric variations were evaluated during the period of IMRT by recalculating and obtaining dose‐volume histograms of re‐contoured target volumes and parotid glands on repeat CT scans taken multiple times during treatment (CT1, CT2, CT3 and CT4).

**Results:**

Result showed significant (*p *< 0.001) mean decrease in both primary and nodal tumors volume with time whereas increase (*p *< 0.01 or *p *< 0.001) in respective V100 (%) and D2% (Gy). The mean parotid gland dose increased (*p *< 0.01 or *p *< 0.001) with time, whereas parotid gland volume and distance between plan isocenter and centre of mass of parotid glands decreased (*p *< 0.05 or *p *< 0.001) with time. Patient's mean weight and neck circumference both decrease (*p *< 0.001) with time whereas ECOG score increase (*p *< 0.001) with time. The mucosal toxicity increased significantly (*p *< 0.001) with time. The change in both weight and neck circumference showed significant (*p *< 0.001) and direct (positive correlation) association with change in parotid gland volume.

**Conclusion:**

If the PTV and normal anatomy are changing with time, adaptive IMRT would be beneficial radiation dose delivery where possible.

## Graphical abstract

Functional impairment and toxicities associated with radiation underscore the need to develop novel treatment strategies in the management of patients with advanced head and neck cancer to improve survival at the cost of minimal toxicity. Conformal radiotherapy techniques such as intensity‐modulated radiotherapy (IMRT) in head neck cancer aids to deliver curative high accuracy radiation doses to the tumor and restricts dose to organs at risk consequently reduce treatment‐related morbidity. However dosimetric variations are evident in IMRT due to variation in patient, tumor volume and organ at risk positions during the course of radiation with risk of radiation overdose.

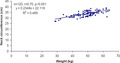

## 1 INTRODUCTION

Conformal radiotherapy techniques such as intensity‐modulated radiotherapy (IMRT) in head and neck cancers (HNC) have allowed radiation oncologists to deliver curative radiation doses to the tumour with higher accuracy while restricting the dose to organs at risk (OARs), consequently reducing treatment‐related morbidity.

However, steep dose gradients are produced in IMRT which imply that there should be no or minimal changes in the patient's anatomy, tumour volume and OARs position so that target volume coverage is not compromised and radiation overdose to critical and normal structures is prevented, thus resulting in enhanced response and reduced radiation toxicity.^1^

Appearance of anatomical variations during the period of radiotherapy in HNC is routinely observed and is due to body weight loss, primary tumour shrinking, parotid gland volume reduction and variation in volume of normal tissue irradiated, which may result in discrepancy in planned dose and actual dose administered causing dosimetric variation of target volume and critical structures with a risk of compromised dose coverage to the target volumes and overdose to the parotid glands and normal tissue influencing treatment response and associated toxicities.^2^, ^3^, ^4^

Therefore, our aim in this study was to evaluate anatomic and volumetric alterations in the parotid glands and tumour volume of HNC patients being treated with IMRT‐SIB, and to study the dosimetric impact of these anatomic changes on dose variation to target volume and parotid glands.

## 2 MATERIALS AND METHODS

30 newly diagnosed, biopsy proven patients with stage II–IV (AJCC Cancer Staging Manual, 8^th^ edition) Head and Neck Squamous Cell Carcinoma (HNSCC) registered at Radiotherapy Department, King George's Medical University, Lucknow India were prospectively enrolled between June 2019 and May 2020. All patients were treated with IMRT step‐and‐shoot modality and received concomitant chemotherapy. Study specific informed consent was taken from all the patients. Study was approved by Institutional Ethics Committee, King George's Medical University. The study was done in accordance with the Declaration of Helsinki and its subsequent amendments, good clinical practice guidelines, and other legal requirements.

Each patient underwent a planning kilo voltage computerized tomography scan (KVCT‐scan) of the head‐and neck region with 3‐mm slice thickness. Patients were scanned in the supine position, immobilized on a flat table top with a customized five fixation points thermoplastic facemask and a head‐and‐neck immobilization board (AIO Board). The planning KVCT images were transferred to a treatment planning system (Monaco Treatment Planning System, Elekta), and contours for the target volumes and normal organs were drawn.

Initial planning CT1 (Plan1) with intravenous contrast agents was acquired from the vertex to the carina. Target volumes and normal structures were manually contoured on the axial slices of the planning CT scan. Gross tumour volume (GTV) was delineated to include primary tumour (GTV‐P) and enlarged neck nodes (GTV‐N) in the enhanced CT images. Three clinical target volume (CTVs), based on the current clinical practice at this institution, were used for each patient: (a) CTV high, which encompassed the GTVs plus a physician‐determined planning margin, was prescribed 66 Gy (at 2.2 Gy per fraction) (b) CTV intermediate, which surrounded the lymph nodes that have a high probability of cancer involvement was prescribed 60 Gy (at 2 Gy per fraction) and (c) CTV low, which encompassed those lymph nodes with a relatively lower probability of cancer involvement and was prescribed 54 Gy (at 1.8 Gy per fraction).

For treatment planning, the PTVs encompassed the CTVs with a 5‐mm margin. The IMRT beam arrangements consisted of seven/nine co‐planar beams. A simultaneous integrated boost technique was used to deliver 66 Gy, 60 Gy and 54 Gy to PTV high, PTV intermediate and PTV low respectively, in 30 fractions over 6 weeks, and the following dose constraints were set on the OAR: maximum dose for the spinal cord, 45 Gy; maximum dose of the brain stem, 54 Gy; mean dose for at least one parotid gland, 26 Gy, although both parotid glands were tried to spare.

All patients received weekly chemotherapy with cisplatin (35mg/m^2^) concurrent with radiotherapy. Patients were weighed and neck circumference of each patient was taken weekly during treatment. Patients were assessed weekly for treatment‐related toxicities. During treatment period, repeat kVCT images with contrast were acquired after patients received 10, 20 and 29 fractions each with the same thermoplastic cast and following the same protocols as during the acquisition of initial CT1 to generate CT2, CT 3 and CT 4. The GTV primary and nodal were delineated as the mass shown in the enhanced CT images. Both the parotid glands were also contoured as seen on the repeat scans of each patient. The initial IMRT plan (Plan 1/CT1) was transferred to CT2, CT3 and CT4 based on carefully matched isocentre and bony alignment to make Plan2, Plan3 and Plan4 respectively. Dose distributions of these plans were recalculated to obtain dose‐volume histograms (DVHs) of re‐contoured target volumes and parotid glands. The changes in volume, distance and dose were analyzed for each patient. To quantify the positional shifts of the parotid glands, we calculated the distance from the centre of mass (COM) of the parotid glands to the matched isocentre for CT scan (CT1, CT2, CT3 and CT4).

Mean dose of the parotid glands and V100, D100%, D98%, and D2% for GTV primary tumor and nodal tumor were evaluated along with anatomical variations in each of these structures on Plan 2/CT2, Plan 3/CT3 and Plan 4/CT4 as compared to initial Plan1/CT1 to assess the effects of anatomic changes on dosimetric variation for each patient during treatment.

Patients were treated as planned on CT1 i.e. Plan 1 and no changes were applied to dose distribution during treatment.

### 2.1 Statistical analysis

Continuous data were summarised in Mean ±SE (standard error of the mean) and compared by repeated measures one‐way analysis of variance (ANOVA) and two‐way ANOVA and the significance of mean difference within and between the groups was done by Newman‐Keuls post hoc test after ascertaining normality by Shapiro‐Wilk's test and homogeneity of variance between groups by Levene's test. Discrete (categorical) groups were summarised in number (n) and percentage (%) and compared by chi‐square (*χ* ^2^) test. Pearson correlation analysis was done to assess association between the variables. A two‐tailed (*α *= 2) *p* < 0.05 was considered statistically significant. Analyses were performed on STATISTICA 7.1 software (StatSoft, Inc.).

## 3 RESULTS AND OBSERVATIONS

The present study assesses the dosimetric impact of anatomical changes occurring in the parotid glands and tumour volume during IMRT‐SIB for HNSCC. A total of 30 patients were recruited and evaluated. Patients were treated with radiotherapy 30 fractions over 6 weeks. The primary outcome measures of the study were primary and nodal tumour related volume and dosimetric variables and volume, mean dose and positional shift of parotid glands. The secondary outcome measures of the study were changes in weight, neck circumference and performance status of patients and correlation between these and the primary outcome measures. All measures were assessed at time of CT1, CT2, CT3 and CT4. We also assessed treatment related acute toxicities in patients during treatment.

### 3.1 Patient's demographic characteristics and tumor details at time of enrolment are summarised in tables 1 and 2

There were 22 male and 8 female patients. The mean (±SE) age of patients was 46.67 ± 1.78 years. The median height, weight, BMI, neck circumference and BSA of patients were 162 cm, 52 kg, 20 kg/m^2^, 35 cm and 1.53 m^2^ respectively. ECOG score of patients ranged from 1–2 with mean 1.13 ± 0.06 and median 1 (Tables 1 and 2).

**TABLE 1:** Characteristics of HNSCC patients at presentation

Variable	No. of patients (n = 30) (%)
Age (years)	46.67 ± 1.78, 25–65, *46*
Gender:	
Female	8 (26.7)
Male	22 (73.3)
Height (cm)	161.86 ± 1.11, 149–178, *162*
Weight (kg)	51.70 ± 1.35, 32–64, *52*
BMI (kg/m^2^)	19.72 ± 0.47, 14–25, *20*
Neck circumference (cm)	34.47 ± 0.43, 29–39, *35*
BSA (m^2^)	1.53 ± 0.02, 1–2, *2*
ECOG (score)	1.13 ± 0.06, 1–2, *1*
Comorbidity	
DM	3 (10.0)
HTN	2 (6.7)
None	25 (83.3)

The age, height, weight, BMI, neck length, neck circumference, BSA and ECOG of patients were summarized in Mean ±SE, range (min‐max) and median respectively whereas gender and co morbidity in number (n) and percentage (%).

Abbreviations: BMI, body mass index; BSA, body surface area of patient; ECOG, Eastern Cooperative Oncology Group Scale of Performance Status.

**TABLE 2:** Tumor details of HNSCC patients at presentation

Variable	No. of patients (n = 30) (%)
Site	
Hypopharynx	4 (13.3)
Larynx	6 (20.0)
Larynx + Hypopharynx	1 (3.3)
Oral cavity	4 (13.3)
Oral cavity + Oropharynx	6 (20.0)
Oropharynx	9 (30.0)
T stage	
1	2 (6.7)
2	16 (53.3)
3	7 (23.3)
4A	5 (16.7)
N stage	
0	11 (36.7)
1	8 (26.7)
2B	3 (10.0)
2C	4 (13.3)
3B	4 (13.3)
M stage	
0	30 (100.0)
Composite stage	
II	5 (16.7)
III	11 (36.7)
IVA	10 (33.3)
IVB	4 (13.3)
SCC differentiation	
Well differentiated	10 (33.3)
Moderately differentiated	14 (46.7)
Poorly differentiated	6 (20.0)

The tumor details of patients were summarized in number (n) and percentage (%).

Most commonly involved site was oropharynx, followed similarly by larynx and oral cavity with oropharynx involvement accounting together for 70.0% of the cases. Patients with stage III and IVA disease made up 70.0% of the study population. 46.7% of the patients had moderately differentiated tumors.

### 3.2 The effect of treatment on patient's weight, neck circumference and ECOG is summarised in table 3

Comparing the mean weight, neck circumference and ECOG score, ANOVA showed significantly different weight (*F* = 28.46, *p* < 0.001), neck circumference (*F* = 16.21, *p* < 0.001) and ECOG score (*F* = 11.00, *p* < 0.001) among the periods (Table 3).

**TABLE 3:** Effect of treatment on weight, neck circumference and ECOG of HNSCC patients over the periods

Variable/Period	Mean ± SE (n = 30)	*F* value	*p* value
Weight (kg)			
CT1	51.70 ± 1.35, 32–64, 52	28.46	<0.001
CT2	50.87 ± 1.33, 31–64, 51		
CT3	48.83 ± 1.34, 29–62, 50		
CT4	47.53 ± 1.32, 29–60, 49		
Neck circumference (cm)			
CT1	34.47 ± 0.43, 29–39, 35	16.21	<0.001
CT2	34.20 ± 0.46, 28–39, 35		
CT3	33.48 ± 0.44, 27–38, 33		
CT4	33.04 ± 0.46, 27–37, 33		
ECOG (score)			
CT1	1.13 ± 0.06, 1–2, 1	11.00	<0.001
CT2	1.10 ± 0.06, 1–2, 1		
CT3	1.13 ± 0.06, 1–2, 1		
CT4	1.43 ± 0.10, 1–3, 1		

The weight, neck circumference and ECOG of patients over the periods were summarized in Mean ± SE, range (min‐max) and median respectively and compared by ANOVA (*F* value).

Abbreviation: ECOG, Eastern Cooperative Oncology Group Scale of Performance Status.

Further, comparing the difference in mean weight, neck circumference and ECOG score between the periods (Table 4), Newman‐Keuls test showed significantly (*p* < 0.01 or *p* < 0.001) different and decreased weight and neck circumference both at CT3 and CT4 as compared to both CT1 and CT2. Furthermore, mean weight also decreased significantly (*p* < 0.05) at CT4 as compared to CT3. In contrast, mean ECOG score increased significantly (*p* < 0.001) at CT4 as compared CT1, CT2 and CT3 but not differ (*p*>0.05) between CT1, CT2 and CT3 i.e. found to be statistically the same.

**TABLE 4:** Comparison (*p* value) of difference in mean weight, neck circumference and ECOG of patients between periods by Newman‐Keuls test

Comparison	Weight (kg)	Neck circumference (cm)	ECOG (score)
CT1 vs. CT2	0.102	0.250	0.618
CT1 vs. CT3	<0.001	<0.001	1.000
CT1 vs. CT4	<0.001	<0.001	<0.001
CT2 vs. CT3	<0.001	0.003	0.872
CT2 vs. CT4	<0.001	<0.001	<0.001
CT3 vs. CT4	0.011	0.056	<0.001

The net mean decrease (i.e. mean change from CT1 to CT4) in weight and neck circumference of patients was found to be 8.1% and 4.1% respectively whereas ECOG score increased by 20.9%.

### 3.3 The effect of treatment on GTV primary tumour related variables [GTV P vol (cc), GTV P V100 (%), GTV P D100% (Gy), GTV P D98% (Gy) and GTV P D2% (Gy)] is summarised in table 5

The mean GTV P vol showed marked decrease with time. Other variables had increased with time.

For each GTV primary tumour related variable, comparing the mean among the periods, ANOVA showed significantly different GTV P vol (*F* = 47.58, *p* < 0.001), GTV P V100 (%) (*F* = 10.76, *p* < 0.001) and GTV P D2 (%) (Gy) (*F* = 4.82, *p*=0.004) (Table 5). However, both GTV P D100 (%) (Gy) and GTV P D98% (Gy) did not showed any significant (*p *> 0.05) change between the periods (Table 5).

**TABLE 5:** Effect of treatment on GTV primary and nodal tumor of patients over the periods

Parameter	Variable/Period	Mean ± SE (n = 30)	*F* value	*p* value
GTV primary tumor	GTV P vol (cc):			
	CT1	29.49 ± 3.54, 1–70, *28*	47.58	<0.001
	CT2	19.66 ± 2.40, 0–46, *17*		
	CT3	14.80 ± 1.98, 0–38, *14*		
	CT4	10.17 ± 1.40, 0–29, *9*		
	GTV P V100 (%):			
	CT1	84.31 ± 2.61, 43–99, *89*	10.76	<0.001
	CT2	74.64 ± 2.75, 47–99, *76*		
	CT3	82.70 ± 3.10, 54–100, *8*4		
	CT4	88.23 ± 2.44, 67–100, *97*		
	GTV P D100% (Gy):			
	CT1	64.31 ± 0.31, 61–68, *64*	1.88	0.139
	CT2	64.09 ± 0.28, 62–68, *64*		
	CT3	64.26 ± 0.28, 62–68, *64*		
	CT4	64.61 ± 0.33, 60–68, *64*		
	GTV P D98% (Gy):			
	CT1	65.96 ± 0.28, 63–70, *66*	1.94	0.129
	CT2	65.67 ± 0.24, 64–69, *66*		
	CT3	65.88 ± 0.24, 63–68, *66*		
	CT4	66.19 ± 0.28, 63–69, *66*		
	GTV P D2% (Gy):			
	CT1	69.17 ± 0.24, 67–73, *69*	4.82	0.004
	CT2	68.97 ± 0.20, 67–73, *69*		
	CT3	69.23 ± 0.19, 67–73, *69*		
	CT4	69.68 ± 0.23, 68–74, 69		
GTV nodal tumor	GTV N vol (cc):			
	CT1	6.00 ± 1.42, 0–29, *5*	13.34	<0.001
	CT2	3.70 ± 0.71, 0–11, *2*		
	CT3	2.06 ± 0.39, 0–6, *1*		
	CT4	1.31 ± 0.27, 0–5, *1*		
	GTV N V100 (%):			
	CT1	49.02 ± 7.65, 0–100, *70*	5.96	0.001
	CT2	51.00 ± 7.93, 0–99, *70*		
	CT3	51.42 ± 8.19, 0–100, *72*		
	CT4	55.79 ± 8.62, 0–100, *85*		
	GTV N D100% (Gy):			
	CT1	40.39 ± 5.71, 0–65, *63*	0.71	0.547
	CT2	40.36 ± 5.71, 0–66, *63*		
	CT3	40.26 ± 5.70, 0–66, *62*		
	CT4	40.71 ± 5.77, 0–67, 64		
	GTV N D98% (Gy):			
	CT1	41.32 ± 5.84, 0–67, *65*	5.42	0.002
	CT2	41.21 ± 5.83, 0–67, *64*		
	CT3	41.34 ± 5.85, 0–69, *64*		
	CT4	41.97 ± 5.93, 0–69, *65*		
	GTV N D2% (Gy):			
	CT1	43.61 ± 6.16, 0–70, *69*	4.88	0.003
	CT2	43.81 ± 6.19, 0–70, *69*		
	CT3	44.58 ± 6.31, 0–78, *69*		
	CT4	44.68 ± 6.32, 0–79, *69*		

Note

The GTV primary and nodal tumor of patients over the periods were summarized in Mean ±SE, range (min‐max) and median respectively and compared by ANOVA (F value)

Further, for each GTV primary tumour related variable, comparing the difference in mean between periods (Table 6), Newman‐Keuls test showed significant (*p* < 0.001) decrease in GTV P vol at CT2, CT3 and CT4 as compared to CT1. Furthermore, it also decreased significantly (*p* < 0.01 or *p* < 0.001) at both CT3 and CT4 as compared to CT2. Moreover, it also decreased significant (*p* < 0.001) at CT4 as compared to CT3. In contrast, GTV P V100 (%) decreased significantly (*p* < 0.01) at CT2 as compared to CT1 but increased significantly (*p* < 0.01 or *p* < 0.001) at both CT3 and CT4 as compared to CT2. Conversely, GTV P D2 (%) (Gy) increased significantly (*p* < 0.05 or *p* < 0.01) at CT4 as compared to CT1, CT2 and CT3 but did not differ (*p *> 0.05) between other period i.e. found to be statistically the same.

**TABLE 6:** Comparison (*p* value) of difference in mean GTV primary tumor and GTV nodal tumor of patients between periods by Newman‐Keuls test

Comparison	GTV primary tumor					GTV nodal tumor				
	GTV P vol (cc)	GTV P V100 (%)	GTV P D100% (Gy)	GTV P D98% (Gy)	GTV P D2% (Gy)	GTV N vol (cc)	GTV N V100 (%)	GTV N D100% (Gy)	GTV N D98% (Gy)	GTV N D2% (Gy)
CT1 vs. CT2	<0.001	0.001	0.570	0.373	0.295	0.005	0.233	0.919	0.590	0.553
CT1 vs. CT3	<0.001	0.515	0.805	0.699	0.749	<0.001	0.320	0.913	0.944	0.017
CT1 vs. CT4	<0.001	0.115	0.192	0.305	0.027	<0.001	0.001	0.333	0.008	0.013
CT2 vs. CT3	0.005	0.002	0.446	0.340	0.358	0.045	0.804	0.761	0.814	0.029
CT2 vs. CT4	<0.001	<0.001	0.099	0.089	0.002	0.011	0.013	0.532	0.003	0.036
CT3 vs. CT4	<0.001	0.069	0.267	0.336	0.023	0.350	0.010	0.515	0.003	0.769

The net mean decrease (i.e. mean change from CT1 to CT4) in GTV Primary volume was 65.5% whereas GTV P V100 (%), GTV P D100% (Gy), GTV P D98% (Gy) and GTV P D2% (Gy) showed 4.4%, 0.5%, 0.3% and 0.7% increase respectively.

### 3.4 The effect of treatment on GTV nodal tumour related variables [GTV N vol (cc), GTV N V100 (%), GTV N D100% (Gy), GTV N D98% (Gy) and GTV N D2% (Gy)] is summarised in table 5

The mean GTV N vol showed marked decrease with time whereas both GTV N V100 and GTV N D2% showed marked increase with time (Table 5).

For each GTV nodal tumour related variable, comparing the mean among periods, ANOVA showed significantly different GTV N vol (*F* = 13.34, *p* < 0.001), GTV N V100 (*F* = 5.96, *p *= 0.001), GTV N D98% (*F* = 5.42, *p *= 0.002) and GTV N D2% (*F* = 4.88, *p *= 0.003) among the periods (Table 5). However, GTV N D100% did not showed any significant (*p *> 0.05) change between the periods (Table 5).

Further, for each GTV nodal tumour variable, comparing the difference in mean between periods (Table 6), Newman‐Keuls test showed significant (*p* < 0.01 or *p* < 0.001) decrease in GTV N vol at CT2, CT3 and CT4 as compared to CT1. It also showed significant (*p* < 0.05) decrease at both CT3 and CT4 as compared to CT2. In contrast, GTV N V100 and GTV N D98% both showed significant (*p* < 0.05 or *p* < 0.01) increase at CT4 as compared to CT1, CT2 and CT3 but found similar (*p*>0.05) between other periods. Conversely, GTV N D2% showed significant (*p* < 0.05) increase at both CT3 and CT4 as compared to both CT1 and CT2 but found similar (*p*>0.05) between CT1 and CT2, and CT3 and CT4 i.e. did not differ significantly.

The net mean decrease (i.e. mean change from CT1 to CT4) in GTV Nodal volume was 78.2% whereas GTV N V100, GTV N D100%, GTV N D98% and GTV N D2% showed increase of 12.1%, 0.8%, 1.5% and 2.4% respectively.

### 3.5 Effect of treatment on the parotid glands

#### 3.5.1 The effect of treatment on the parotid gland which received higher mean dose at planning on CT1 relative to contralateral side [H‐Parotid gland *D*_mean_ (Gy), H‐Parotid gland volume (cc) and Distance between plan isocenter and COM of H‐Parotid gland (cm)] is summarised in table 7

**FIGURE 1:**
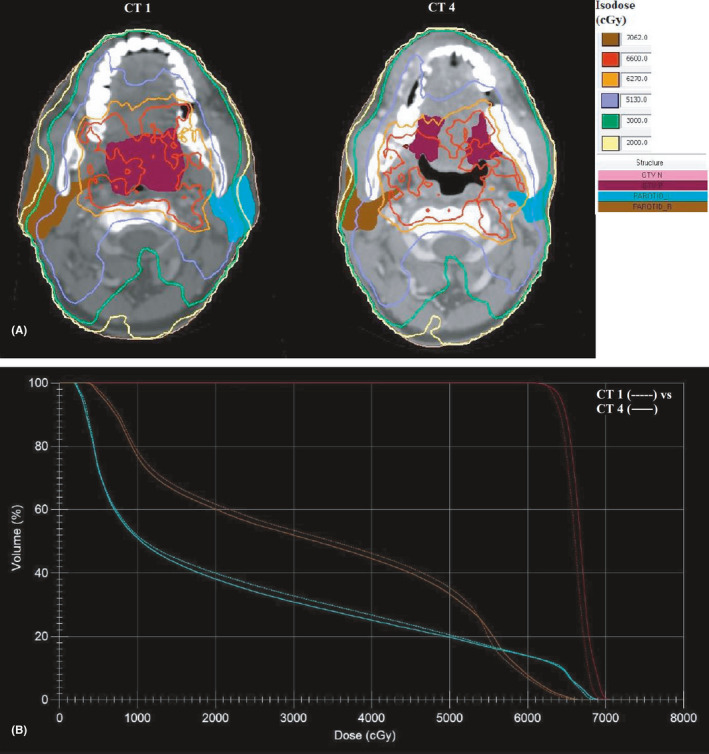
A, Axial CT sections of a T2N0 soft palate carcinoma. Dose distribution of Plan 1 on CT1 (left) and on CT4(right). Patient lost about 6 kgs during treatment. The parotid glands decreased in size during treatment. The 30 Gy (light green) and 20 Gy (light yellow) isodose lines shifted closer to lateral border of the parotid glands in CT4 compared to CT1. B,. DVH comparison between CT1(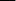) and CT4 (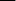)

**FIGURE 2:**
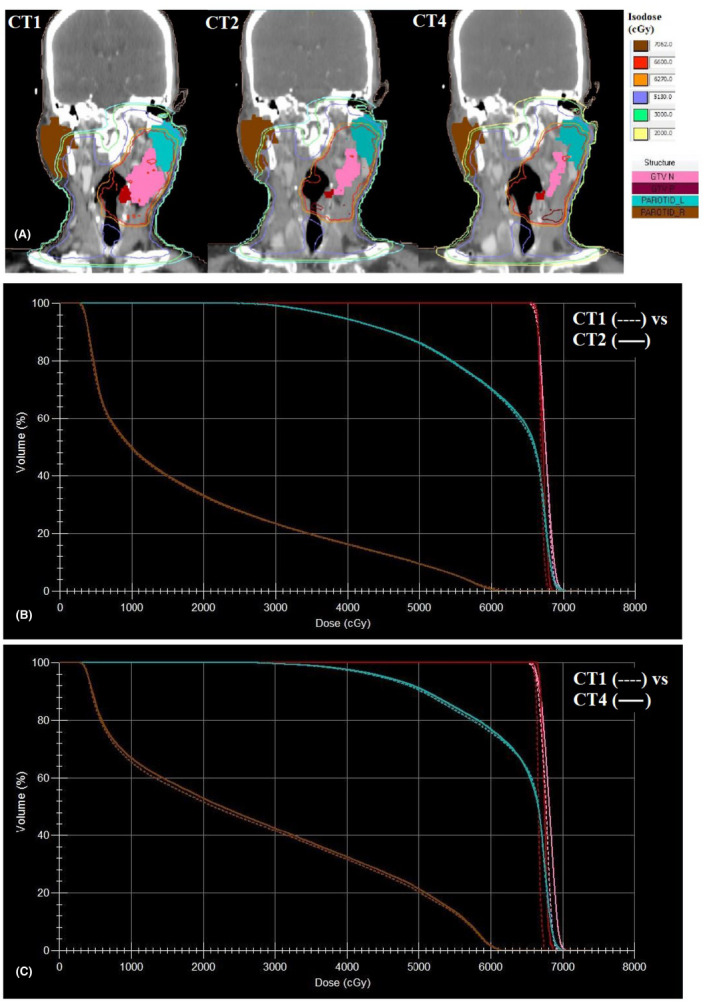
A, Coronal CT sections of a 55‐years‐old carcinoma pyriform fossa (T1N3b) patient. The gradual decrease in GTV primary (maroon), GTV nodal (pink) and bilateral parotid gland volumes can be appreciated. The low dose isodose curves [20 Gy (light yellow) and 30 Gy (light green)] can be seen covering more areas of right parotid gland in CT4 compared to CT1 and CT2. b. Comparative DVH of CT1 (‐‐‐‐‐‐‐) and CT2 (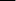) c. Comparative DVH of CT1 (‐‐‐‐‐‐‐) and CT4 (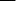)

The mean H‐Parotid gland *D*_mean_ showed linear increase with time whereas both H‐Parotid gland volume and distance between plan isocenter and COM of H‐Parotid gland showed linear decrease with time (Figures 1 and 2).

For each H‐Parotid gland related variable, comparing the mean among periods, ANOVA showed significantly different H‐Parotid gland *D*_mean_ (*F* = 16.51, *p* < 0.001), H‐Parotid gland volume (*F* = 91.77, *p* < 0.001) and distance between plan isocenter and COM of H‐Parotid gland (*F* = 26.50, *p* < 0.001) (Table 7).

**TABLE 7:** Distribution of H‐Parotid glands, L‐Parotid glands and Both‐Parotid glands of HNSCC patients over the periods

Parameter	Variable/Period	Mean ± SE (n = 30)	*F* value	*p* value
Higher dose parotid glands	H‐Parotid gland *D*_mean_ (Gy):			
	CT1	38.28 ± 1.97, 19–61, 38	16.51	<0.001
	CT2	38.37 ± 2.15, 22–62, 37		
	CT3	38.81 ± 2.23, 20–62, 38		
	CT4	41.28 ± 2.05, 28–63, 38		
	H‐Parotid gland volume (cc):			
	CT1	29.24 ± 1.14, 17–38, 30	91.77	<0.001
	CT2	24.83 ± 1.05, 12–34, 27		
	CT3	21.27 ± 1.03, 9–33, 22		
	CT4	20.02 ± 1.08, 7–31, 20		
	Distance between plan isocenter and COM of H‐Parotid gland (cm)			
	CT1	5.12 ± 0.09, 4–6, 5	26.50	<0.001
	CT2	4.97 ± 0.09, 4–6, 5		
	CT3	4.71 ± 0.10, 3–6, 5		
	CT4	4.65 ± 0.09, 4–6, 5		
Lower dose parotid glands	L‐Parotid gland *D*_mean_ (Gy):			
	CT1	25.38 ± 1.61, 18–45, 21	4.49	0.006
	CT2	25.46 ± 1.67, 15–45, 22		
	CT3	26.39 ± 1.78, 17–49, 22		
	CT4	27.52 ± 1.61, 16–48, 25		
	L‐Parotid gland volume (cc):			
	CT1	29.15 ± 1.28, 16–37, 33	84.13	<0.001
	CT2	25.81 ± 1.22, 13–35, 28		
	CT3	22.49 ± 1.18, 11–33, 24		
	CT4	20.37 ± 1.31, 7–32, 21		
	Distance between plan isocenter and COM of L‐Parotid gland (cm):			
	CT1	4.90 ± 0.10, 4–6, 5	28.90	<0.001
	CT2	4.80 ± 0.09, 4–6, 5		
	CT3	4.67 ± 0.10, 4–6, 5		
	CT4	4.53 ± 0.10, 4–6, 4		
Both dose parotid glands	BOTH‐Parotid glands *D*_mean_ (Gy):			
	CT1	31.89 ± 1.51, 18–47, 31	2.40	0.073
	CT2	32.20 ± 1.52, 22–47, 31		
	CT3	33.12 ± 1.65, 20–48, 33		
	CT4	33.72 ± 1.51, 24–51, 31		
	BOTH‐Parotid glands volume (cc):			
	CT1	64.04 ± 2.48, 37–79, 68	107.83	<0.001
	CT2	56.92 ± 2.35, 30–74, 59		
	CT3	49.60 ± 2.27, 25–67, 52		
	CT4	46.43 ± 2.42, 19–66, 48		
	Distance between plan isocenter and center of mass (COM) of BOTH‐Parotid glands (cm):			
	CT1	0.41 ± 0.05, 0–1, 0	3.82	0.013
	CT2	0.33 ± 0.05, 0–1, 0		
	CT3	0.31 ± 0.04, 0–1, 0		
	CT4	0.31 ± 0.05, 0–1, 0		

Note

The H‐Parotid& L‐Parotid gland variables of patients over the treatment period were summarized in Mean ± SE, range (min‐max) and median respectively and compared by ANOVA (*F* value).

Further, for each H‐Parotid gland related variable, comparing the difference in mean between periods (Table 8), Newman‐Keuls test showed significant (*p* < 0.001) increase in H‐Parotid gland *D*_mean_ at CT4 as compared to other periods whereas it was found to be statistically the same (*p *> 0.05) between other periods. In contrast, both H‐Parotid gland volume and distance between plan isocenter and COM of H‐Parotid gland showed significant (*p* < 0.05 or *p* < 0.001) decrease at CT2, CT3 and CT4 as compared to CT1. Both variables also showed significant (*p* < 0.001) decrease at both CT3 and CT4 as compared to CT2. The H‐Parotid gland volume showed significant (*p* < 0.05) decrease at CT4 as compared to CT3.

**TABLE 8:** Comparison (*p* value) of difference in mean higher, lower and both dose parotid glands of patients between treatment periods by Newman‐Keuls test

Comparison	Higher dose parotid glands			Lower dose parotid glands			Both dose parotid glands		
	[H] Parotid Dose *D*_mean_ (Gy)	[H] Parotid vol (cc)	Distance between plan isocenter and COM of H‐Parotid gland (cm)	[L] Parotid Dose *D*_mean_ (Gy)	[L] Parotid vol (cc)	Distance between plan isocenter and COM of L‐ Parotid gland (cm)	[BOTH] Parotid Dose *D*_mean_ (Gy)	[BOTH] Parotid vol (cc)	Distance between plan isocenter and center of mass (COM) of BOTH‐Parotid glands (cm):
CT1 vs. CT2	0.845	<0.001	0.012	0.900	<0.001	0.018	0.687	<0.001	0.021
CT1 vs. CT3	0.532	<0.001	<0.001	0.288	<0.001	<0.001	0.249	<0.001	0.022
CT1 vs. CT4	<0.001	<0.001	<0.001	0.010	<0.001	<0.001	0.088	<0.001	0.015
CT2 vs. CT3	0.382	<0.001	<0.001	0.168	<0.001	0.004	0.234	<0.001	0.824
CT2 vs. CT4	<0.001	<0.001	<0.001	0.008	<0.001	<0.001	0.123	<0.001	0.607
CT3 vs. CT4	<0.001	0.043	0.294	0.094	0.001	0.001	0.437	0.004	0.938

At final evaluation the H‐Parotid gland shrank in volume by 31.6% and shifted medially by 9.2% from CT1 to CT4 with a net mean increase in *D*_mean_ of 7.3%.

#### 3.5.2 The effect of treatment on the parotid gland which received lower mean dose at planning on CT1 relative to contralateral side [L‐Parotid gland *D*_mean_ (Gy), L‐Parotid gland volume (cc) and Distance between plan isocenter and COM of L‐Parotid gland (cm)] is summarised in table 7

The mean L‐Parotid gland *D*_mean_ showed linear increase with time whereas both L‐Parotid gland volume and Distance between plan isocenter and COM of L‐Parotid gland showed linear decrease with time (Figures 1 and 2).

For each L‐Parotid gland variable, comparing the mean among periods, ANOVA showed significantly different L‐Parotid gland *D*_mean_ (*F* = 4.49, *p *= 0.006), L‐Parotid gland volume (*F* = 84.13, *p* < 0.001) and distance between plan isocenter and COM of L‐Parotid gland (*F *= 28.90, *p* < 0.001) among the periods (Table 7).

Further, for each L‐Parotid gland variable, comparing the difference in mean between periods (Table 8), Newman‐Keuls test showed significant (*p* < 0.05 or *p* < 0.01) increase in L‐Parotid gland *D*_mean_ at CT4 as compared to CT1, CT2 and CT3 whereas it was found to be statistically the same (*p *> 0.05) between CT1, CT2 and CT3. In contrast, both L‐Parotid gland volume and distance between plan isocenter and COM of L‐Parotid gland showed significant (*p* < 0.05 or *p* < 0.001) decrease at CT2, CT3 and CT4 as compared to CT1. Both variables showed significant (*p* < 0.01 or *p* < 0.001) decrease at both CT3 and CT4 as compared to CT2. Further, both variables also showed significant (*p* < 0.01) decrease at CT4 as compared to CT3.

At final evaluation the L‐Parotid gland shrank in volume by 30.1% and shifted medially by 7.5% from CT1 to CT4 with a net mean increase in *D*_mean_ of 7.8%.

#### 3.5.3 The effect of treatment on variables related to combined volume of both parotid glands of the patient [BOTH‐Parotid glands *D*_mean_ (Gy), BOTH‐Parotid glands volume (cc) and Distance between plan isocenter and COM of BOTH‐Parotid glands (cm)] is summarised in table 7

The mean BOTH‐Parotid glands *D*_mean_ showed linear increase with time whereas BOTH‐Parotid glands volume and distance between plan isocenter and COM of BOTH‐Parotid glands showed linear decrease with time.

For each, BOTH‐parotid glands related variable, comparing the mean among periods, ANOVA showed significantly different BOTH‐Parotid glands volume (*F* = 107.83, *p* < 0.001) and Distance between plan isocenter and COM of BOTH‐Parotid glands (*F* = 3.82, *p* < 0.05) among the periods (Table 7). However, BOTH‐Parotid glands *D*_mean_ showed insignificant change among the periods (*F* = 2.40, *p *=0.073).

Further, for each, BOTH‐parotid glands related variables, comparing the difference in mean between periods (Table 8), Newman‐Keuls test showed significant (*p* < 0.05 or *p* < 0.001) decrease in BOTH‐Parotid glands volume and distance between plan isocenter and COM of BOTH‐Parotid glands at CT2, CT3 and CT4 as compared to CT1. Furthermore, BOTH‐Parotid glands volume also showed significant (*p* < 0001) decrease at both CT3 and CT4 as compared to CT2. Moreover, it also showed significant (*p* < 0.01) decrease at CT4 as compared to CT3.

At final evaluation, BOTH‐Parotid glands *D*_mean_ showed net mean increase (i.e. mean change from CT1 to CT4) of 5.4% whereas BOTH‐Parotid glands volume and Distance between plan isocenter and COM of BOTH‐Parotid glands showed net mean decrease of 27.5% and 23.8% respectively.

### 3.6 Correlation

**FIGURE 3:**
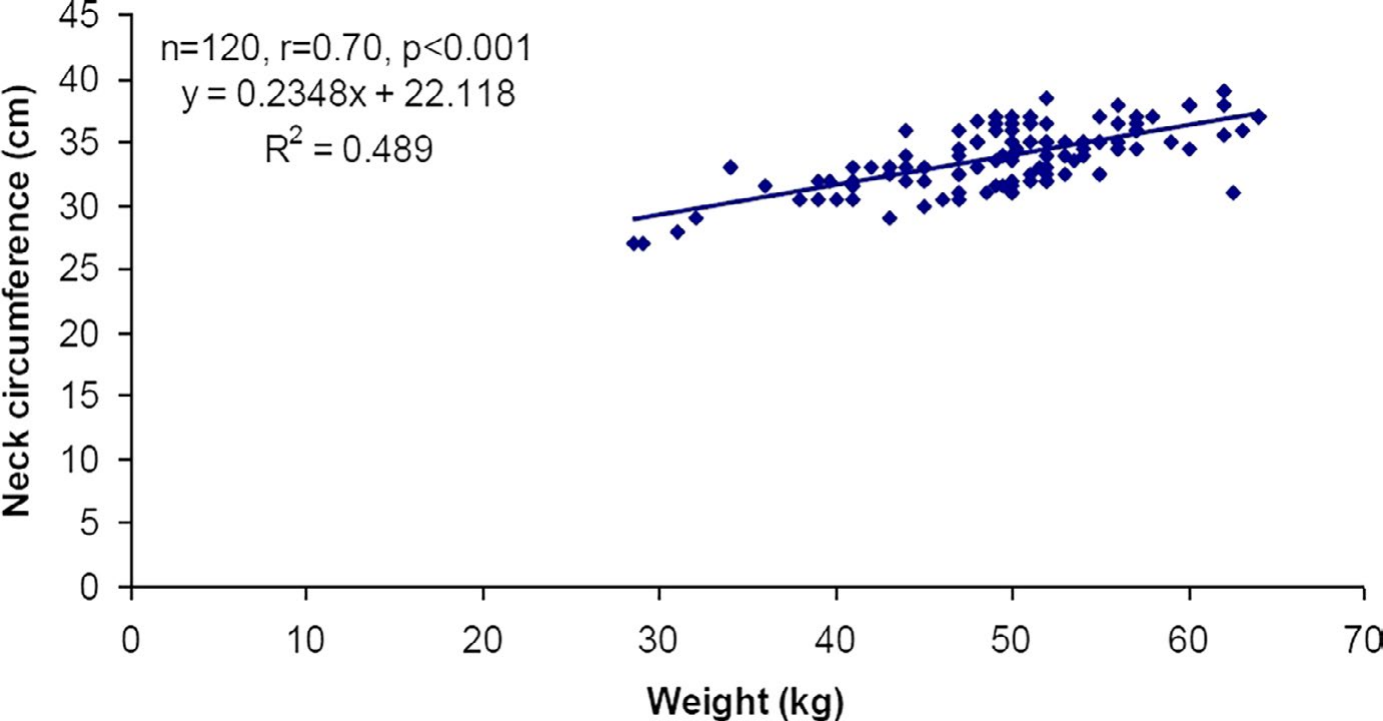
Correlation between changes in weight and neck circumference over the time

**FIGURE 4:**
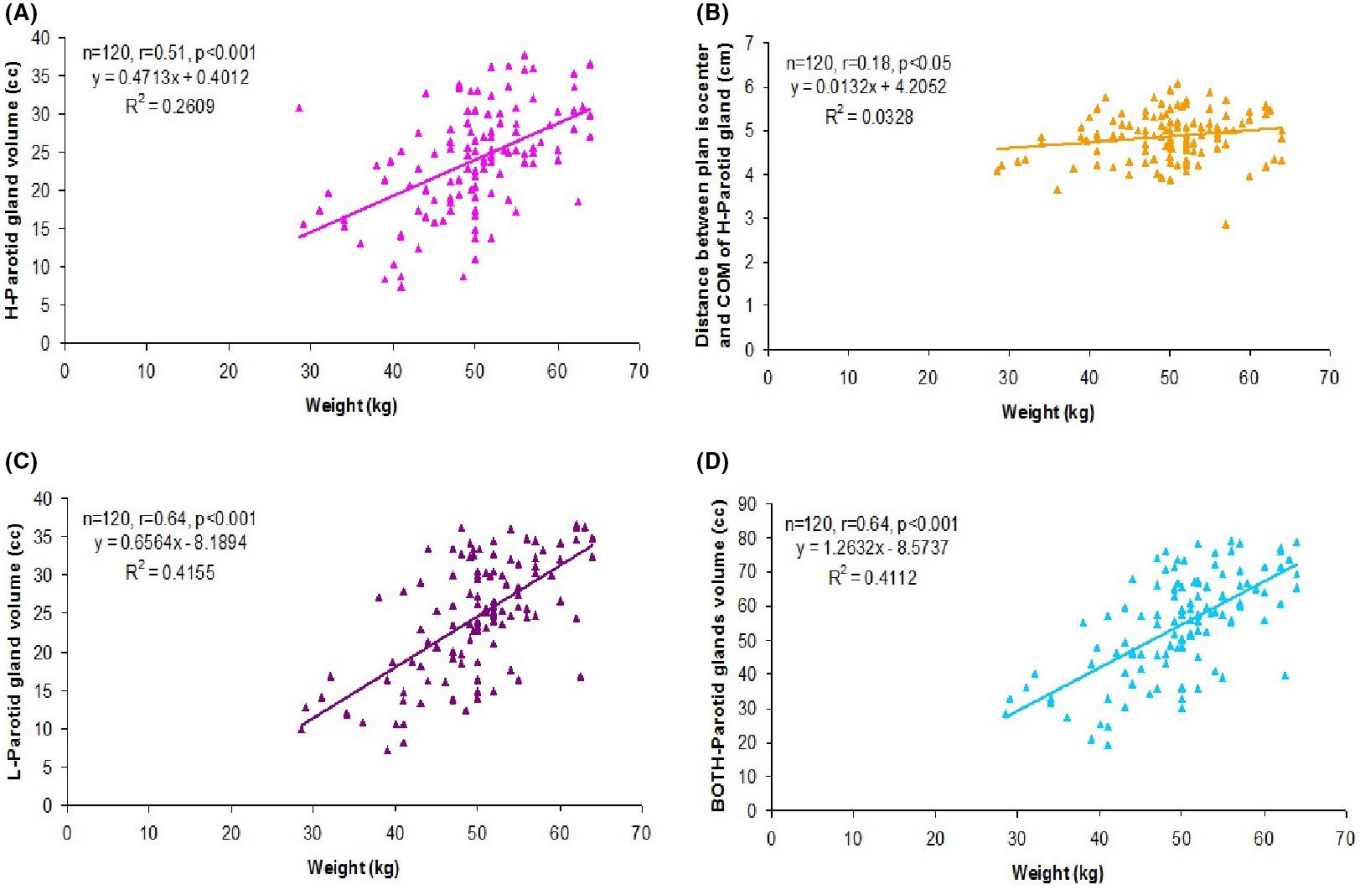
(A) Correlation between weight and H‐Parotid gland volume over the time (B) Correlation between weight and distance between plan isocenter and COM of H‐Parotid gland over the time (C) Correlation between weight and L‐Parotid gland volume over the time (D) Correlation between weight and BOTH‐Parotid glands volume over the time

**FIGURE 5:**
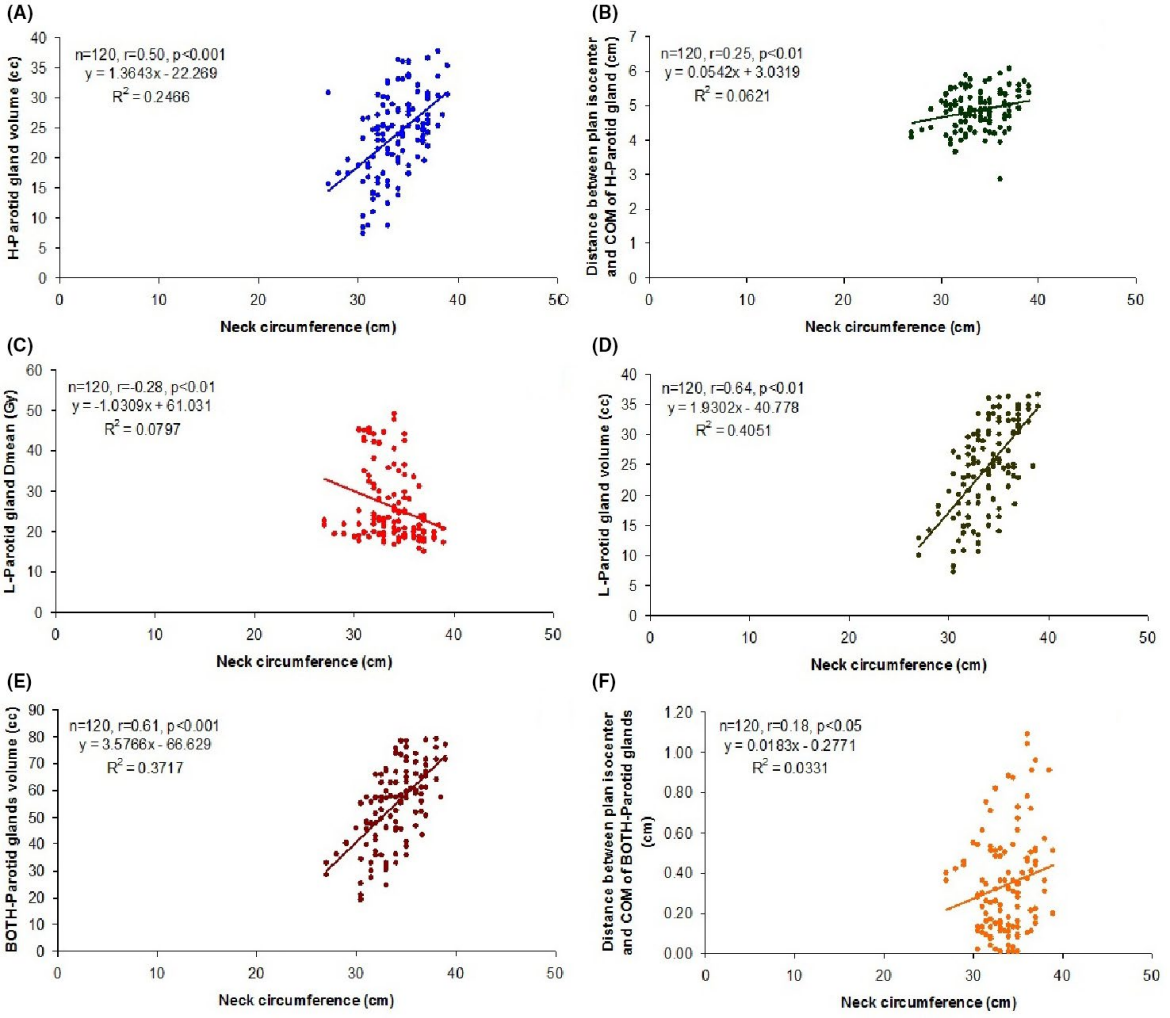
(A) Correlation between neck circumference and H‐Parotid gland volume over the time (B) Correlation between neck circumference and distance between plan isocenter and COM of H‐Parotid gland over the time (C) Correlation between neck circumference and L‐Parotid gland *D*_mean_ over the time (D) Correlation between neck circumference and L‐Parotid gland volume over the time (E) Correlation between neck circumference and BOTH‐Parotid glands volume over the time (F) Correlation between neck circumference and distance between plan isocenter and COM of BOTH‐Parotid glands over the time

The correlation of change in both weight and neck circumference with change in parotid gland (*D*_mean_, volume and distance) of patients over the periods (CT1+CT2+CT3+CT4, n = 120) is summarised in Table 9. The Pearson correlation analysis showed a significant and positive (direct) correlation between change in neck circumference and change in weight of patients (*r* = 0.70, *p *< 0.001) (Table 9 and Figure 3). Further, change in H‐Parotid gland volume (*r* = 0.51, *p *< 0.001), Distance between plan isocenter and COM of H‐Parotid gland (*r* = 0.18, *p *< 0.05), L‐Parotid gland volume (*r* = 0.64, *p *< 0.001) and BOTH‐Parotid glands volume (*r* = 0.64, *p *< 0.001) showed a significant and positive correlation with change in weight (Table 9 and Figure 4A–D). In contrast, change in H‐Parotid gland volume (*r* = 0.50, *p *< 0.001), Distance between plan isocenter and COM of H‐Parotid gland (*r* = 0.25, *p *< 0.01), L‐Parotid gland volume (r=0.64, *p *< 0.001), BOTH‐Parotid glands volume (*r* = 0.61, *p *< 0.001) and Distance between plan isocenter and COM of BOTH‐Parotid glands (*r* = 0.18, *p *< 0.05) showed a significant and positive correlation whereas L‐Parotid gland *D*_mean_ (*r* = −0.28, *p *< 0.01) showed a significant and negative (inverse) correlation with change in neck circumference (Table 9 and Figure 5A–F).

**TABLE 9:** Inter‐correlation between response of different variables of HNSCC patients over the periods (n = 120)

Variable	Weight	Neck circumference	H‐Parotid gland *D*_mean_	H‐Parotid gland volume	Distance between plan isocenter and COM of H‐Parotid gland	L‐Parotid gland *D*_mean_	L‐Parotid gland volume	Distance between plan isocenter and COM of L‐Parotid gland	BOTH‐Parotid glands *D*_mean_	BOTH‐Parotid glands volume	Distance between plan isocenter and COM of BOTH‐Parotid glands
Weight	1.00										
Neck circumference	0.70***	1.00									
H‐Parotid gland *D*_mean_	0.07**^ns^**	0.08**^ns^**	1.00								
H‐Parotid gland volume	0.51***	0.50***	−0.11**^ns^**	1.00							
Distance between plan isocenter and COM of H‐Parotid gland	0.18*	0.25**	0.18*	0.15**^ns^**	1.00						
L‐Parotid gland *D*_mean_	−0.11**^ns^**	−0.28**	0.34***	−0.43***	−0.17**^ns^**	1.00					
L‐Parotid gland volume	0.64***	0.64***	0.00**^ns^**	0.89***	0.19*	−0.41***	1.00				
Distance between plan isocenter and COM of L‐Parotid gland	0.17**^ns^**	0.09**^ns^**	0.00**^ns^**	0.16**^ns^**	−0.20*	−0.16**^ns^**	0.07**^ns^**	1.00			
BOTH‐Parotid glands *D*_mean_	−0.04**^ns^**	−0.10**^ns^**	0.83***	−0.26**	0.01**^ns^**	0.73***	−0.19*	−0.05**^ns^**	1.00		
BOTH‐Parotid glands volume	0.64***	0.61***	−0.05**^ns^**	0.92***	0.16**^ns^**	−0.41***	0.97***	0.10**^ns^**	−0.25**	1.00	
Distance between plan isocenter and COM of BOTH‐Parotid glands	0.00**^ns^**	0.18*	−0.19*	0.18*	0.24**	−0.16**^ns^**	0.14**^ns^**	−0.19*	−0.22*	0.15**^ns^**	1.00

ns‐ *p* >0.05,

* *p* < 0.05,

** *p* < 0.01,

*** *p* < 0.0.

### 3.7 Toxicity during treatment

The distribution of maximum grade (RTOG) of toxicity (haematological, skin, salivary gland and mucosal) that occurred in patients during treatment showed Grade 2 haematological toxicity in 13 (43.3%) patients. Grade 2 and 3 skin toxicity was found in 23 (76.7%) and 3 (10.0%) patients respectively. Grade 2 and 3 mucosal toxicity was seen in 17 (56.7%) and 8 (26.7%) patients respectively. 16 (53.3%) patients required nasogastric tube insertion during treatment to maintain adequate nutrition. During radiotherapy, salivary gland Grade 1 toxicity was seen in 3 (10.0%) patients whereas 27 (90%) patients had Grade 2 toxicity.

The mucosal toxicity of patients at the time of repeat scans showed that the higher‐grade toxicity (>2) in patients increased significantly with time (*χ* ^2^ = 79.84, *p* < 0.001).

## 4 DISCUSSION

IMRT in the HNC was specifically introduced to minimize irradiation of the parotid glands and to improve the patient's quality of life after radiotherapy.^5^

In the present study, the patients experienced a significant decrease in weight and neck circumference after having received twenty fractions of radiotherapy. Moreover, decrease in neck circumference was significantly associated with decrease in weight. We found a significant correlation between decrease in patient's weight with decrease in volume of both the parotid glands as well as medial shift of the parotid gland which received higher mean dose at initial planning. Decrease in neck circumference correlated well with decrease in volume of both parotid glands and their medial shift as well as increase in mean dose to the parotid gland which received lower mean dose at initial planning. The reduction of the head thickness leads consequently to the occurrence of dose hotspot in the neck, close or within the parotid glands as observed by Castelli et al.^6^ You et al found that patients with significant reduction of the neck diameter and/or weight loss showed significantly frequent grade 2 acute xerostomia.^7^

9 patients were seen to have a decline in ECOG status, mostly after the fourth week of treatment. Decline in performance status was also noted in a study by Lohia *et al*.^8^ This could be associated with IMRT related fatigue and other treatment related toxicities.

We observed a decrease in the volumes of GTV P and GTV N by 65.5% and 78.2% respectively. Similarly, Barker *et al* reported a median total relative loss of 69.5% of the initial GTV on the last day of treatment.^2^ The amount of normal mucosa around the gross tumour volume that needs to be included in the clinical target volume is unclear, but even in the IMRT era most primary‐tumour failures typically occur in the gross tumour volume and not in the surrounding mucosal area.^9^

Dosimetric coverage of the target volumes tends to be robust during radiotherapy. The current study found no difference in GTV P D98% from start to end of treatment, while there was a slight but significant increase in GTV P D2%, GTV N D98% and GTV N D2%. Wu *et al*. reported no change in the delivered dose to the primary CTV, with small a small increase in the minimum dose delivered to the nodal CTV, likely caused by the larger volume and anatomic changes experienced by the nodal CTV.^10^ Similarly, Nishi *et al* also reported a slight increase in dose to the primary GTV in their study of 20 patients who underwent a repeat CT scan partway through treatment. They reported no changes in the minimum delivered dose to the nodal GTV.^4^ Castadot *et al* who also investigated the impact of anatomic changes on target coverage reported that the dose to the primary and nodal CTVs remained unchanged as a result of anatomic changes throughout radiotherapy.^11^

This study showed that the parotid glands decreased in volume by about 30% by end of treatment. Likewise, Bhide *et al* and Ho et al reported a contraction of the parotid gland volumes by 35% and 25% respectively through the course of treatment.^12^, ^13^

The medial shift of parotid glands on either side and the linear increase in their mean dose with time as observed in our study, correlated well with other published literature.^2^, ^4^, ^6^, ^10^, ^11^, ^12^, ^14^, ^15^ The anatomic changes observed over time, as quantified in this study, are particularly important, because the parotid glands move medially towards the high‐dose region (Figure 1 and 2). This implies that most of the radiation dose was delivered to a deviated anatomy compared with the original treatment plan.

Despite advancements in the RT technique, acute toxicities continue to be a major challenge in HNC radiotherapy. Mucositis and xerostomia were the most common acute toxicities seen in our patients. The strength of this study is that we have taken multiple CT images of the same patient during treatment period in the treatment position and compared these with the initial simulation images with respect to the anatomic changes of bilateral parotid glands and the primary as well as nodal tumor volumes to assess the dosimetric changes on the same. We have also correlated these changes with change in patient's weight loss and changes in neck circumference. We have also monitored the acute treatment related toxicities. Limitation of this study is that in view of limited resources, patients had not undergone midcourse replanning to compensate for the anatomical changes that they underwent, it may have resulted in optimum dose distributions and reduced long term toxicities for some patients.

## 5 CONCLUSION

With temporally changing anatomy of both tumour and normal tissue, delivery of radiotherapy should be temporally changing to match the observed anatomic changes where possible, however, needs further validation on larger population.

## AUTHOR CONTRIBUTIONS

Arunima Ghosh: Collected Data, Contributed in paper writing Seema Gupta: Formulated and designed the manuscript and analysis, Collected Data, Contributed in data and data analysis tools, Contributed in paper writing Danial Johny: Contributed in data and data analysis tools Vivek Vidyadhar Bhosale: Contributed in paper writing Mahendra Pal Singh Negi: Contributed in data and data analysis tools, Performed data analysis.

## MESSAGE OF THE MANUSCRIPT

If the planning target volume and normal tissue anatomy are changing with time during IMRT, adaptive IMRT would be beneficial radiation dose delivery where possible to minimize normal tissue toxicity without influencing therapeutic outcome.

## Conflict of interest

There is none.

## Data Availability

Data available on request from the authors.
